# Transcriptome Analysis to Understand the Toxicity of *Latrodectus tredecimguttatus* Eggs

**DOI:** 10.3390/toxins8120378

**Published:** 2016-12-20

**Authors:** Dehong Xu, Xianchun Wang

**Affiliations:** 1 Key Laboratory of Protein Chemistry and Developmental Biology of Ministry of Education, College of Life Sciences, Hunan Normal University, Changsha 410081, China; 2 Laboratory of Biological Engineering, College of Pharmacy, Hunan University of Chinese Medicine, Changsha 410208, China; xudehong163@126.com

**Keywords:** *Latrodectus tredecimguttatus*, eggs, Illumina sequencing, proteinaceous toxin, transcriptome

## Abstract

*Latrodectus tredecimguttatus* is a kind of highly venomous black widow spider, with toxicity coming from not only venomous glands but also other parts of its body as well as newborn spiderlings and eggs. Up to date, although *L. tredecimguttatus* eggs have been demonstrated to be rich in proteinaceous toxins, there is no systematic investigation on such active components at transcriptome level. In this study, we performed a high-throughput transcriptome sequencing of *L. tredecimguttatus* eggs with Illumina sequencing technology. As a result, 53,284 protein-coding unigenes were identified, of which 14,185 unigenes produced significant hits in the available databases, including 280 unigenes encoding proteins or peptides homologous to known proteinaceous toxins. GO term and KEGG pathway enrichment analyses of the 280 unigenes showed that 375 GO terms and 18 KEGG pathways were significantly enriched. Functional analysis indicated that these unigene-coded toxins have the bioactivities to degrade tissue proteins, inhibit ion channels, block neuromuscular transmission, provoke anaphylaxis, induce apoptosis and hyperalgesia, etc. No known typical proteinaceous toxins in *L. tredecimguttatus* venomous glands, such as latrotoxins, were identified, suggesting that the eggs have a different toxicity mechanism from that of the venom. Our present transcriptome analysis not only helps to reveal the gene expression profile and toxicity mechanism of the *L. tredecimguttatus* eggs, but also provides references for the further related researches.

## 1. Introduction

*Latrodectus tredecimguttatus* is a kind of black widow spider, belonging to Arthropoda, Arachnoidea, Araneida, Theridiidae, and Latrodectus [1]. Such spiders are found in much of the world including Central Asia, Southern Europe [2] and Xinjiang, Yunnan, Inner Mongolia and Gansu Provinces, China [3]. *L. tredecimguttatus* spiders are highly poisonous and usually cause adverse reactions even lethal effects on humans and animals by envenomation [4], so ordinary people are afraid of the spiders while toxinologists are interested in them because all kinds of toxic components contained in poisonous spiders constitute a library of natural bioactive substances to discover and screen for pharmacological tool reagents, drug leads and insecticides [5,6,7]. As one of the most toxic spider species, the black widow spider differs from snake, scorpion and some other venomous spider species in that it has toxic components not only in its venomous glands [8,9,10,11] but also throughout its body, even in its newborn spiderlings and eggs [12,13,14]. Our research group has been using chromatography, proteomics, patch clamp and other related techniques to comprehensively analyze spider egg toxicity. The results showed that the proteinaceous components in the egg extract are mainly high-molecular-mass proteins and the peptides below 5 kDa, and that the toxicity of the eggs are mainly due to the high-molecular-mass proteins [14,15]. Besides, three high-molecular-mass proteins and one low-molecular-mass peptide, named latroeggtoxin-I to latroeggtoxin-IV, respectively, were purified and characterized from the egg extract. Among them, Latroeggtoxin-I reversibly blocked the neuromuscular transmission in isolated mouse phrenic nerve-hemidiaphragm preparations; latroeggtoxin-II selectively inhibited tetrodotoxin-resistant (TTX-R) sodium channel currents in rat dorsal root ganglion neurons (DRG), without significant effect on the tetrodotoxin-sensitive (TTX-S) sodium channel currents; latroeggtoxin-III showed inhibitory effects on the potassium and calcium channel currents in cockroach dorsal unpaired median (DUM) neurons; and latroeggtoxin-IV was shown to be a broad-spectrum antibacterial peptide [16,17,18].

Although much effort has been devoted to discovering and characterizing active proteinaceous components in spider eggs, many such components have been left out due to the low expression abundance in translation level and the low sensitivity of the methods used. In view of this situation, we are in urgent need of a new method to sensitively and efficiently investigate the proteinaceous components for revealing egg toxicity.

In recent years, with the development of next-generation sequencing technology especially RNA sequencing, transcriptome research has made considerable progress and is now widely applied in discovering and identifying proteinaceous toxins from venomous animals. Illumina/Solexa is one of the most commonly used RNA high-throughput sequencing technologies and has developed Hiseq2000, Hiseq2500 and other sequencing platforms. Compared with traditional expressed sequence tags (EST) sanger sequencing, Illumina/Solexa has many incomparable advantages, such as high throughput, low cost and high sensitivity, while it can detect low abundance of gene expression [19]. In addition, the development of bioinformatics including sequence assembly software and proteinaceous toxin databases of main venomous animals provides great convenience for assembly de novo and functional annotation of the unigenes without prior genome information, and also promotes the continuous development and deepening of the transcriptome study in venomous animals [20,21,22].

In our present study, we made a transcriptome analysis of the *L. tredecimguttatus* eggs. The unigenes assembled de novo were annotated, categorized and analyzed using bioinformatics methods, which provides a systematic insight into the toxicity of the eggs at transcriptome level.

## 2. Results

### 2.1. Illumina Sequencing and Read Assembly

The cDNA library constructed by mRNA reverse transcription of *L. tredecimguttatus* eggs was sequenced using Illumina Hiseq^TM^ 2500 PE125. After the sequencing quality filtering step, a total of 47,970,296 reads composed of 5,836,590,233 bp were obtained, each with an average length of 121 bp. Using paired-end joining and gap-filling, these sequencing reads were de novo assembled with software Trinity (version: trinityrnaseq_r2013-02-25) followed by de-redundance. As a result, 69,684 non-redundant transcripts (contigs) with lengths greater than 200 bp were produced, from which 53,284 unigenes (accounting for 76.47%) were obtained following the principle that if several transcripts with different sizes belonged to the same unigene, the longest one was chosen. The average length of the unigenes was 738 bp, ranging from 201 bp to 19,858 bp. Among the 53,284 unigenes, 4376 (8.21%) were greater than 2000 bp and 14,185 (26.62%) were annotated in at least one of the available databases (Table 1 and Figure S1). Raw sequencing data could be downloaded from SRA of NCBI using the accession number SRP068948.

### 2.2. Categories and Annotations of Unigenes

**Figure 1:**
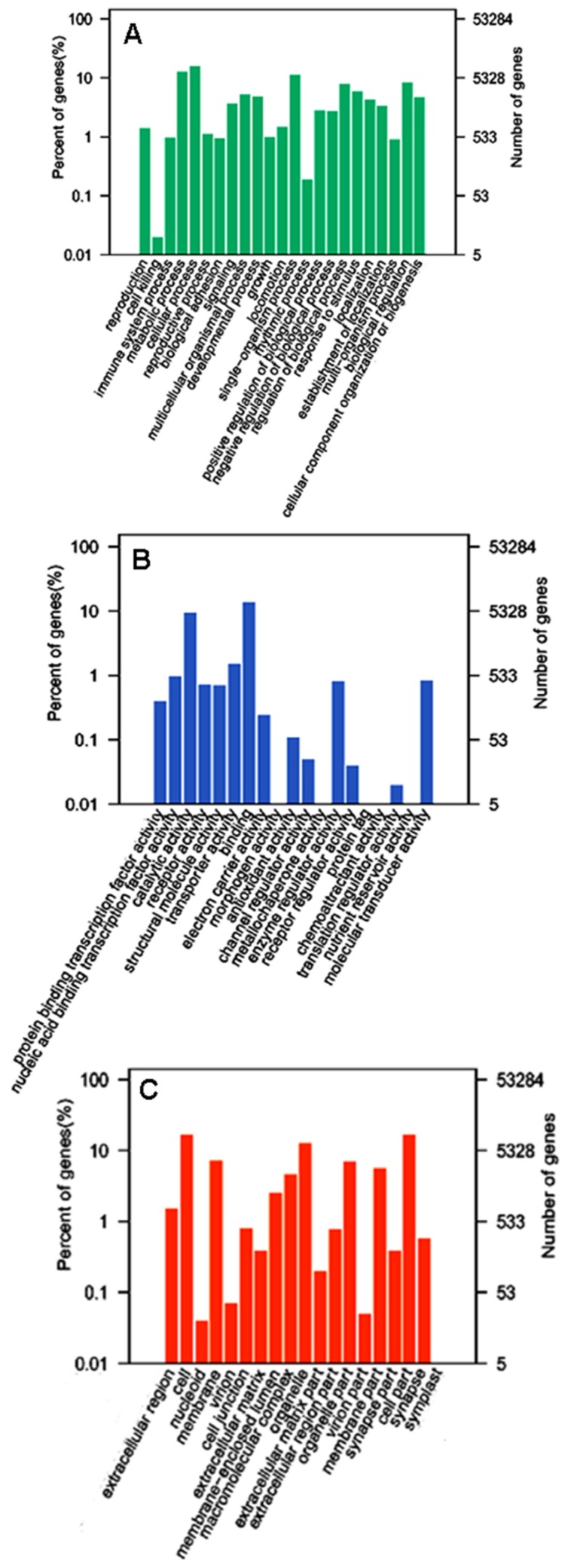
GO classification of the unigenes from *L. tredecimguttatus* eggs. Unigenes were annotated within three categories: biological process (**A**); molecular function (**B**); and cellular component (**C**). The *x*-axis represents the different categories. The number and percent of the unigenes matching GO annotation terms are presented on the *y*-axis.

To systematically analyze the characteristics of the unigenes, Gene Onthology (GO) annotation of the unigenes was firstly carried out. On the basis of GO annotation results, 11,630 unigenes/proteins were annotated within three main GO ontologies, namely biological process, cellular component and molecular function. The GO analysis regarding biological process revealed two major gene categories of cellular process and metabolic process, followed by those related to single-organism process, biological regulation, response to stimulus, cellular component organization or biogenesis, multicellular organismal process, development process, localization, etc. (Figure 1A). The analysis in relation to the molecular function showed that the proteins with binding function and catalytic activity were the most representative components (Figure 1B). Besides, analysis of cellular component indicated that the identified proteins were distributed in various parts of the cell, including the cell interior and the cell surface (Figure 1C). These data suggest that proteins identified through the transcriptome analysis are extensively distributed in various subcellular locations of the *L. tredecimguttatus* eggs and involved in multiple cellular processes and biological functions, particularly in binding, catalysis, development, etc., which is in agreement with the basic functions of the eggs and the results of our previous work [14,15].

**Figure 2:**
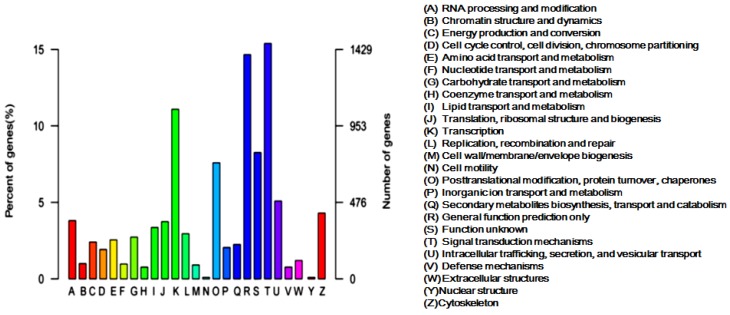
KOG classification of the egg unigenes: 9528 annotated unigenes were classified into 25 KOG categories. The *x*-axis represents the different KOG categories. The number and percent of unigenes matching KOG classification are presented on the *y*-axis.

**Figure 3:**
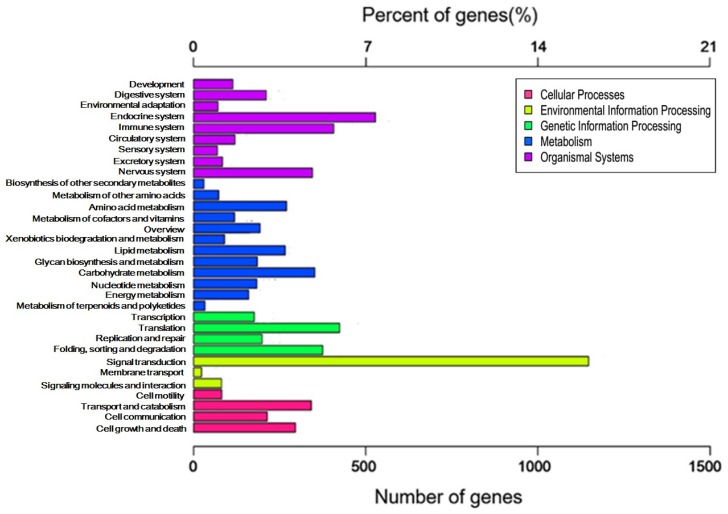
KEGG classification of the egg unigenes: 3141 unigenes were classified into 317 pathways. The different colors represent different KEGG categories. The number and percent of the unigenes matching KEGG classification are presented on the upper and lower axes, respectively.

To further evaluate the obtained unigenes, we searched against the Clusters of Orthologous Groups (KOG) database for KOG classification of the unigenes. A total of 9528 unigenes were functionally divided into 25 categories, of which the category “Signal transduction mechanisms” presented the largest group (1466, 14.92%), followed by “General function prediction only” (1397, 14.66%) and “Transcription” (1057, 11.09%). The clusters for “Cell motility and nuclear structure” presented the smallest groups (Figure 2). For identifying the biological pathways active in the *L. tredecimguttatus* eggs, we made the Kyoto Encyclopedia of Genes and Genomes (KEGG) annotation of the unigenes. As a result, 3141 unigenes were classified into 317 pathways. Pathways in cancer (ko05200) was the most dominant pathway with 141 unigenes, followed by Protein processing in endoplasmic reticulum (ko04141), Ribosome (ko03010), RNA transport (ko03013) and Huntington's disease (ko05016), which contained 129, 129, 128, and 125 unigenes, respectively. The identified pathways were involved in metabolism, organismal systems, environmental information processing, genetic information processing, and cellular processes. Signal transduction in the group of environmental information processing contained the largest number of the unigenes (1147) assigned into the pathways (Figure 3). These data on the categories and annotations of the unigenes provide a valuable reference for investigating the composition, structure and function of the proteins and the toxicity basis of *L. tredecimguttatus* eggs.

### 2.3. GO and KEGG Pathway Enrichment Analyses of Unigenes

**Figure 4:**
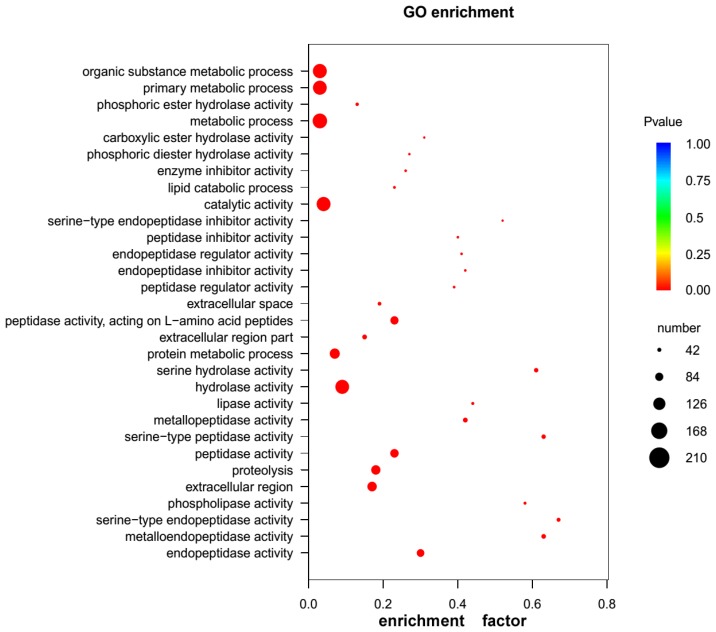
GO enrichment analysis scatterplot for the 280 unigenes encoding toxins. The size of black circle represents the unigene number. Different colors represent different *p* values for significance test.

**Figure 5:**
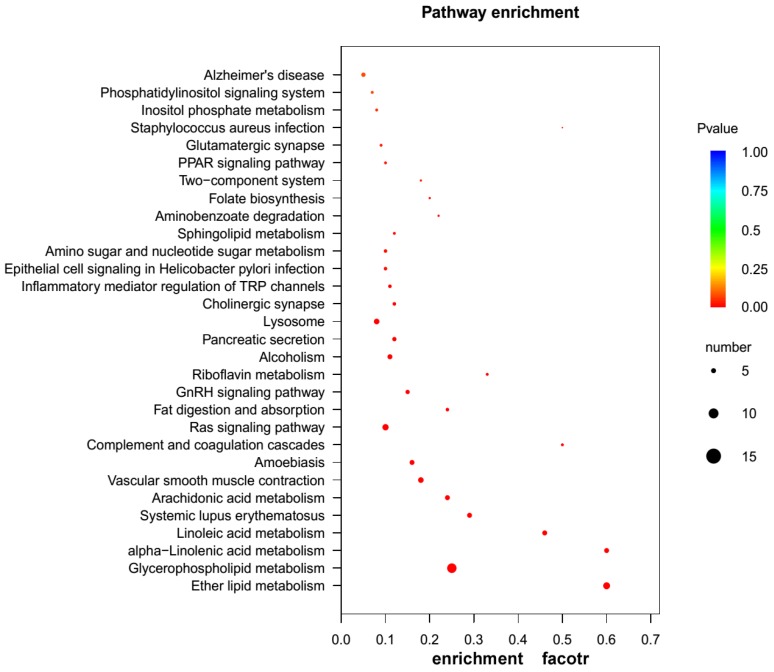
KEGG enrichment analysis scatterplot for the 280 unigenes encoding toxins. The size of black circle represents the unigene number. Different colors represent different *p* values for significance test.

Following the procedures to mine proteinaceous toxins from the eggs described in the Materials and Methods Section, it was found that among the 14,185 unigenes annotated in at least one of the available databases, 280 (1.97%) unigenes encoded the proteins or peptides homologous to the known proteinaceous toxins. To further elucidate their functional roles in the toxicity of the eggs, we performed GO enrichment analysis for the 280 unigenes. The results showed that 262 unigenes had GO annotations, of which 375 GO terms were significantly enriched, with 75 terms in molecular function (MF), 25 terms in cellular component (CC) and 275 terms in biological process (BP) (FDR < 0.05, Table S1). Most of the terms in BP were enriched in organic substance metabolic process, primary metabolic process and metabolic process, whereas the highly enriched terms in MF were hydrolytic enzyme activity and enzyme inhibitor/regulator activity, such as peptidase activity, metallopeptidase activity, serine hydrolase activity, phospholipase activity, etc. (Figure 4 and Table S1), which suggest that the eggs toxicity is closely related to the actions of the hydrolytic enzymes in the eggs. In order to further understand the toxicity mechanism of the eggs, we conducted KEGG pathway enrichment analysis for the 280 unigenes. In total, 70 unigenes were mapped to 100 KEGG pathways, and 18 of these KEGG pathways were significantly enriched (FDR < 0.05, Table S2). The top three KEGG pathways were glycerophospholipid metabolism (ko00564), ether lipid metabolism (ko00565) and Ras signaling pathway (ko04014), respectively (Figure 5 and Table S2). Notably, two KEGG pathways, cholinergic synapse (ko04725) and glutamatergic synapse (ko04724), were involved in the nerve system especially nerve impulse conduction in the organismal systems. These results suggest that the unigenes enriched in these KEGG pathways may exert toxic effects by interfering with normal transmission of neurotransmitter in the victims, and that these proteins in the eggs may be closely involved in the neural system development of spider embryos. As for other unigenes enriched in metabolism pathways, although not directly linked to the egg toxicity, they may form potential regulatory networks involved in toxic determination.

### 2.4. Proteinaceous Toxins in the Eggs

**Figure 6:**
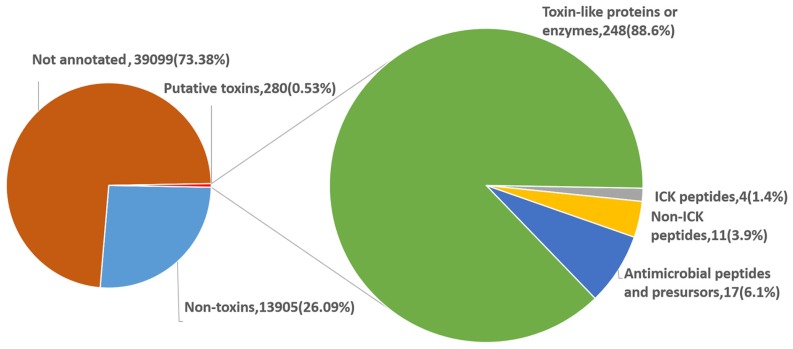
Distribution of the egg unigenes. Left pie graph shows the composition of the egg unigenes. “Not annotated” represents the unigenes not annotated in the available databases. Unigenes annotated to encode the proteins matching known toxins are labeled as “Putative toxins”, and those matching other proteins are labeled as “Non-toxins”. The number of the unigenes in each subcategory is given followed by its percentage shown in the bracket. Right pie graph is further classification of the putative toxins. Putative toxins are further divided into four types. The number of the unigenes in each type is given followed by its percentage shown in the bracket. ICK, inhibitor cystine knot.

Based on structure and function, the proteinaceous toxins encoded by the 280 unigenes were categorized into four types: ICK motif peptide toxins, non-ICK motif peptide toxins, antimicrobial peptides and precursors, and toxin-like proteins or enzymes. As the most abundant components, toxin-like proteins or enzymes accounted for about 88.6% of the proteinaceous toxins in the eggs (Figure 6).

#### 2.4.1. ICK Motif Peptide Toxins

**Figure 7:**
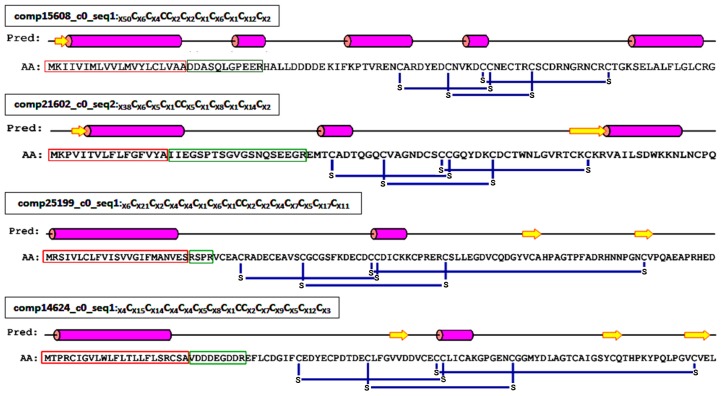
Primary and secondary structure analyses of the predicted ICK toxins. The amino acids forming an alpha helix are colored in pink. Yellow arrows represent β-sheet. Red and green rectangles indicate predicted signal peptides and propeptides, respectively. Blue lines show the connecting pattern of disulfide bonds.

**Figure 8:**
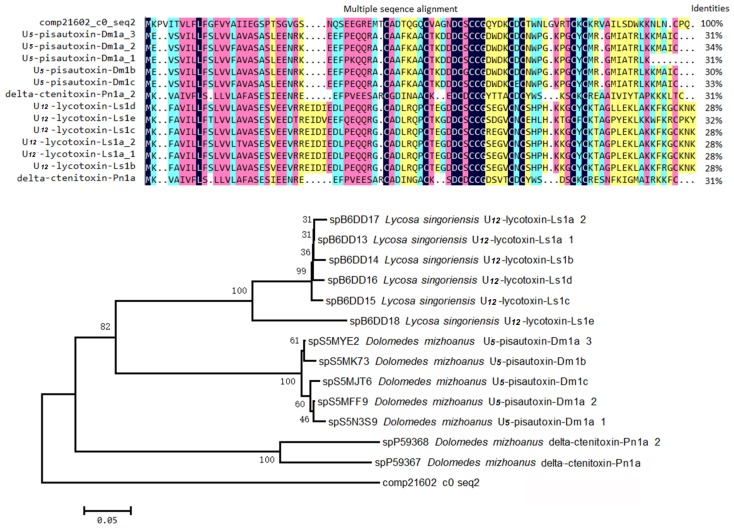
Sequence alignment and phylogenetic analysis of comp21602_c0_seq2. Alignment was performed by DNAman soft. Strictly conserved cysteins are indicated by deep blue and other less conserved amino acids are marked using different colors. Phylogenetic analysis was implemented in MEGA 3.1 using the neighbor-joining method. ArachnoServer accession numbers precede the species name for each sequence. Numbers at the nodes indicate the bootstrap values based on 10,000 replicates.

**Figure 9:**
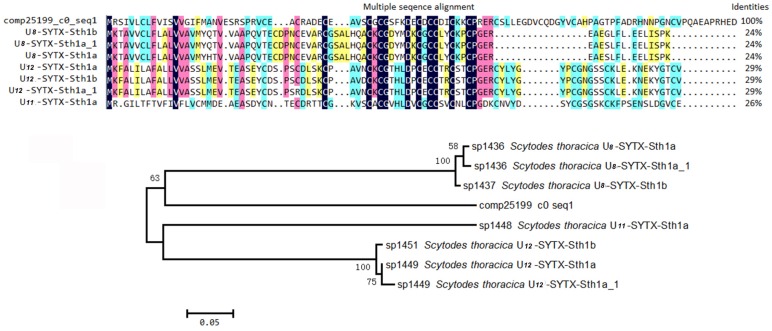
Sequence alignment and phylogenetic analysis of comp25199_c0_seq1. Alignment was performed by DNAman soft. Strictly conserved cysteins are indicated by deep blue and other less conserved amino acids are marked using different colors. Phylogenetic analysis was implemented in MEGA 3.1 using the neighbor-joining method. ArachnoServer accession numbers precede the species name for each sequence. Numbers at the nodes indicate the bootstrap values based on 10,000 replicates.

Inhibitor cystine knot (ICK) is a topologically structural motif that contributes to the strong stability and exceptional function of the peptides that contain it. For example, the vast majority of peptides containing ICK motif in spider venom have neurotoxic activities [23,24]. To date, the venoms of two kinds of black widow spiders (*L. tredecimguttatus* and *L. hesperus*) have been reported to contain such neurotoxic peptides [8,25]. Therefore, it is reasonable to speculate that there are ICK motif peptides in the *L. tredecimguttatus* eggs. By mining the transcriptome data of *L. tredecimguttatus* eggs, we discovered four ICK motif peptides containing signal peptides and propeptides in their N-terminus (Figure 7 and Table S3), which is consistent with the structural feature of the neurotoxic peptides in spider venom [26,27]. When aligning the four ICK motif peptides with the toxins in ArachnoServer database by BLAST at an E-value cut-off of 10^−5^ (E-value < 0.00001), we found that two of them had significant hits. One ICK motif peptide (comp25199_c0_seq1) was homologous with the toxins from *Scytodes thoracica* and the other (comp21602_c0_seq2) was homologous with the toxins from three different spider species, namely *Dolomedes mizhoanus*, *Lycosa singoriensis* and *Phoneutria nigriventer*, though their sequence identities were not very high (24%–34%). When we further carried out multiple sequence alignment and phylogenetic tree analysis for above two ICK motif peptides and the homologous toxins in the database, we found that although their cysteine arrangements were highly conserved, the two ICK motif peptides were located on the independent clades of the two phylogenetic trees, respectively, which indicated that the genetic relationship between the ICK motif peptides and the homologous toxins in the database is not too close. Therefore, there may be obvious differences in their functions, which need to be studied by further experiments (Figure 8 and Figure 9).

#### 2.4.2. Non-ICK Motif Peptide Toxins

Just as the peptide toxins in spider venom are various in their types [28,29], predicted peptide toxins from the eggs include not only ICK motif peptide toxins but also some other non-ICK motif peptide toxins. Eleven non-ICK motif peptides were found in the egg transcriptome by BLAST analysis at E-value < 10^−5^ (Table 2 and Table S3). The unigene (comp21232_c0_seq1) encodes a non-ICK motif peptide that shares the highest identity (98%) with the cystatin-like protein from the venom of *Latrodectus hesperus*, another kind of black widow spider. Cystatins are cysteine-protease inhibitors and present in a wide range of organisms such as vertebrates, invertebrates, and plants as well as protozoa [30,31]. For example, the venom of reptilia snakes and saliva of arachnida ticks were reported to contain cystatin as a toxic component [32,33]. Cystatins are involved in multiple biological processes, e.g., antigen presentation, immune system development, epidermal homeostasis, neutrophil chemotaxis during inflammation, or apoptosis [32,34]. It was reported that cystatin in snake venom can protect proteinaceous toxins from degradation of prey proteases, and in tick saliva is involved in blood feeding and transmitting various pathogens by its immunosuppressive properties. Our present transcriptome analysis demonstrated that the eggs of *L. tredecimguttatus* contain cystatin and this bioactive peptide was speculated to play roles in, for example, protecting and assisting other proteinaceous components to exert their biological activities, just like the cystatins in elapid snake and other venomous organisms [32,33,34].

Three of the non-ICK motif peptides (comp20935_c0_seq1, comp213809_c0_seq1 and comp27859_c0_seq1) have homology with U_24_-ctenitoxin-Pn1a, a toxin from the venom of spider *Phoneutria nigriventer*. According to the functional annotation in Swiss-Prot database, U_24_-ctenitoxin-Pn1a contains TY domain and may act as a protease inhibitor. U3-aranetoxin-Ce1a from the spider *Caerostris extrusa* is predicted to be a neurotoxic peptide in Swiss-Prot database, so its homologous peptide (comp5553_c0_seq1) in the eggs probably has the same function. Another three peptides (comp17051_c0_seq1, comp25199_c0_seq1 and comp96908_c0_seq1) are homologous with three toxins from other spider species with unknown molecular target. Therefore, their functions need to be verified by further experiments. In addition, three peptides (comp24914_c0_seq1, comp22890_c0_seq2 and comp6833_c0_seq1) are homologous with toxin-like peptides or proteins from scorpion venom. Such homology was also found in the analyses of venom gland transcriptomes of *L. tredecimguttatus* and *S. thoracica* [8,29], which supports the viewpoint to some extent that in the long evolutionary process of arachnids, some ancient peptides as toxic molecules had been recruited repeatedly in different arachnids.

#### 2.4.3. Antimicrobial Peptides and Precursors

**Figure 10:**
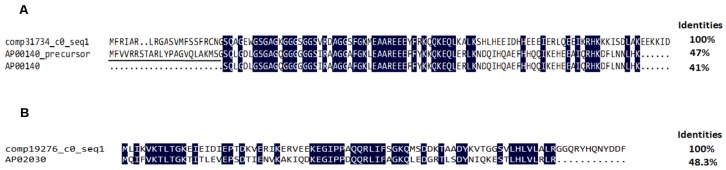
Amino acid sequence alignment of comp31734_c0_seq1 and comp19276_c0_seq1: (**A**) Multisequence alignment of comp31734_c0_seq1 with AP00140 and its precursor (GenBank:GJ19999). The underlined amino acid residues indicate a putative signal peptide sequence. (**B**) Sequence alignment of comp19276_c0_seq1 with AP2030. Identical residues are shaded in blue in both (**A**) and (**B**).

*L. tredecimguttatus* lives in diverse environments, such as caves, farmland, and drains. Therefore, it needs to face a variety of environmental tests for survival, of which resistance to pathogenic microorganisms is a common test. As a class of arthropods, spiders rely mainly on humoral immunity in innate defenses to resist infection by pathogenic microorganisms. Among the humoral immune factors, there is growing evidence showing that antimicrobial peptides (AMPs) are the most important components of innate immune defenses [35]. To date, although more than 40 AMPs have been isolated from different araneomorph spiders [36], AMPs from *L. tredecimguttatus*, a member of araneomorphs, have been less reported. In this study, we searched the unigenes from *L. tredecimguttatus* eggs against the antimicrobial peptides database (APD) using BlastX with an E-value threshold of 10^−5^ [37]. As a result, it was found that seventeen peptides have homology with two AMPs and four major classes of antimicrobial peptide precursors in the database (Table S3). The unigene (comp31734_c0_seq1) encodes a peptide consisting of 112 amino acid residues and sharing 41% identity with an AMP (APD ID: AP00140) in the antimicrobial peptides database (Figure 10A). AP00140 is a novel glycine-rich peptide isolated from the larvae of *Drosophila virilis* and its precursor is a pseudoprotein. The functional research of AP00140 showed that it had specific inhibitory effects on the tested Gram-positive bacteria and cancer cell lines, but had no hemolytic activity [38]. In the same way, another peptide encoded by unigene (comp19276_c0_seq1) was found to match an AMP (AP02030) from an acidified gill extract of *Crassostrea gigas* with a sequence identity of 48.3% (Figure 10B). AP02030 is an ubiquitin lacking the terminal Gly-Gly doublet and ending in a C-terminal Arg residue which might be related to antimicrobial activity against Gram-positive and Gram-negative bacteria without hemolytic activity [39]. Based on the high similarity between above two AMPs from antimicrobial peptides database and the corresponding peptides from *L. tredecimguttatus* eggs, it is reasonable to predict that the two peptides found in the eggs might also have antimicrobial activity.

The types and structures of AMPs in many organisms are diverse [40]. Some AMPs may be present in different organisms in the form of precursors. There are increasing reports that some common proteins in cells, such as histone 2A, ubiquitin, thrombin, glyceraldehyde-3-phosphate dehydrogenase (DAPDH), can be considered as the potential antimicrobial peptide precursors, because their N-terminus or C-terminus often form peptides with antimicrobial activity after the degradation by different proteases in vivo [39,41,42,43,44]. In this study, the results from the egg transcriptome showed that there were unigenes to encode four major classes of antimicrobial peptide precursors, histone 2A, ubiquitin, thrombin and DAPDH. Besides, the data from our present work indicated that various proteases were abundant in the eggs (see below). Therefore, it can be supposed that once eggs are infected by pathogenic microorganisms, AMPs will be released from the precursors by different proteases and, together with other components in innate immune defenses, resist pathogenic microorganism.

#### 2.4.4. Toxin-Like Proteins or Enzymes

Li et al. [15] have used proteomics strategy to identify proteins of the *L. tredecimguttatus* eggs and compared egg proteome with venom gland venom proteome. They found that the protein composition of the eggs is more complex than that of venomous gland venom and the eggs are rich in enzymes. In addition, the molecular weights of the proteins in eggs are mainly distributed in the range of from about 34 kDa to above 170 kDa, with the highest abundant protein bands at around 65 kDa and 130 kDa, respectively. Besides, Yan et al. [14] studied the physiological and biochemical properties of the egg extract, and the results showed that the extract could completely block the neuromuscular transmission in mouse isolated phrenic nerve-hemidiaphragm preparations and inhibit the voltage-activated Na^+^, K^+^ and Ca^2+^ currents in rat DRG neurons. Furthermore, the neurotoxicity of the eggs to mammals was shown to be primarily attributed to their high-molecular-mass protein components. Identification and characterization of these proteins are useful to thoroughly understand the toxicity mechanism of the eggs. In this research, toxin-like proteins or enzymes discovered from the eggs are listed in Table 3.

**Metalloproteases and serine proteases.** Sixty-two unigenes encoding metalloproteases were discovered in the eggs (Table S3), accounting for 25% of the toxin-like proteins or enzymes. Therefore, metalloproteinases are high-abundant toxin-like enzymes. Further analysis showed that metalloproteases in the eggs belong to the zinc metalloprotease family, including astacin-like metalloproteases, disintegrin and metalloproteinase with thrombospondin motifs, etc. Astacin-like metalloproteases are one of the important proteolytic enzymes, which have been found in a wide variety of spider venoms [29,45]. Ten unigenes were identified to encode astacin-like metalloproteases in the eggs, of which one unigene (comp30635_c0_seq1) encodes a whole protein and another two unigenes (comp105515_c0_seq1 and comp26980_c0_seq1) lacking start or termination codon encode two long protein fragments. Multisequence alignment analysis for the proteins encoded by the above three unigenes revealed that although they are members of the astacin family with the consensus sequence HEXXHXXGXXHE and Met-turn motif SXMXY (X is any amino acid; Figure S2), the deduced amino acid sequences of other regions in the proteins are not conservative, which indicates that astacin-like metalloproteases in the eggs are of different types.

Serine proteases constitute another main group of proteolytic enzymes in the eggs. A total of 55 unigenes were identified to encode these enzymes (Table S3), whose molecular structure and catalyzing substrate had diversity [62]. Just like the proteases in spider venoms [48], the metalloproteases and serine proteases in the eggs may play important roles in the activation of proteinaceous toxins by cleaving precursors, such as the hydrolysis of antimicrobial peptide precursors. Besides, these proteases may degrade prey tissues and thus facilitate the spreading of toxins. In addition, they may also be involved in the toxicity to protect the eggs from predators, because they could prevent predators from continuously eating eggs by destroying their normal tissues and causing bleeding [46,47,48].

**Phospholipases.** Forty-one unigenes were found to encode phospholipases in the eggs (Table S3), of which two unigenes (comp12124_c0_seq1 and comp121968 _c0_seq1) encode phospholipases that have homology with the phospholipase D from *Loxosceles* genus spider venom. Comp12124_c0_seq1 and comp121968_c0_seq1 share 41% and 53% identity with LrSicTox-alphaIB1 and LiSicTox-betaID1, two phospholipase D isoforms, respectively. The *Loxosceles* genus spider venoms are complex mixtures of toxins especially enriched in phospholipase D, which may cause dermonecrosis, hemolysis, thrombocytopenia, etc. [47,48]. Therefore, we can speculate that phospholipase D may also be an important ingredient to cause toxicity of the *L. tredecimguttatus* eggs. Besides phospholipase D, another phospholipase endowing eggs with toxicity may be phospholipase A2 that accounts for 34% of total phospholipases in the eggs, because it has been reported to present in a variety of animal venoms as a toxin exhibiting various pathological activities, including cytotoxicity and neurotoxicity [49,57,63].

**Serine protease inhibitors (Serpins).** Serpins are one of the most important protease inhibitors, which play roles as potential toxins and are widely distributed in the venoms of poisonous animals, such as snake, scorpion, spider and so on. The mechanisms by which serpins act and exert their noxious effects are not fully clear, but it has been proposed that one of the putative roles is to protect their venom toxins from prey proteases [46] and another putative role is to regulate the activity of voltage-gated ion channels. For example, HWTX-XI from spider and Hg1 from scorpion are two Kunitz-type and bi-functional toxins because they are trypsin inhibitors as well as Kv1.1 and Kv1.3 potassium channel blockers, respectively [47,50,51]. In the eggs, there are 30 unigenes (Table S3) to encode serpins, of which five unigenes (comp10510_c0_seq1, comp199008_c0_seq1, comp191339_c0_seq1, comp31304_c0_seq2 and comp15360_c0_seq1) encode Kunitz-type serpins, suggesting that the inhibitory activity of the egg extract on voltage-gated ion channels especially potassium channels described by Yan et al. [14] may involve these components.

**Phosphatases.** Phosphatases are the enzymes that hydrolyze phosphate esters non-specifically and ubiquitously present in the venom of various poisonous animals, such as snake, spider, wasp and so on [5,64,65]. The toxinological properties of phosphatases in venoms have been less characterized, but, recently, there have been some reports suggesting that these enzymes in snake venom could either endogenously liberate purines, which act as a multitoxin, or synergistically act with other toxins, contributing to the overall lethal effects of the venoms [52]. In our present research, 18 unigenes were found to encode phosphatases (Table S3), which were speculated to not only participate in substance and energy metabolism in the eggs, but also play important roles in the egg toxicity.

**Cholinesterases.** Cholinesterase is a protease to block the transmission of information between nerve and muscle by hydrolyzing neurotransmitter acetylcholine [53], and is often present in the venom of snakes as a neurotoxic component [66]. For *L. tredecimguttatus*, cholinesterase activity has been found in the venom but not in the eggs [67,68]. In this study, 12 unigenes (Table S3) encoding such enzymes were discovered in the eggs for the first time, which were shown to be homologous with the cholinesterase from Stegodyphus mimosarum by BLASTX analysis. Combining with the previous physiological and biochemical characterization of the egg extract by Yan et al. [14], we conjecture that the inhibitory effects of egg extract on the neuromuscular transmission may be at least partially related to the cholinesterases in the egg extract.

**Allergens/Lipocalins.** Allergens/Lipocalins have been identified as potential toxins in spider venoms, which are potent molecules to cause sensitization and disrupt hemostasis [29,46,54,55,56]. Data from our present work showed that 11 unigenes in the *L. tredecimguttatus* eggs encoded allergens/lipocalins (Table S3). Although the functions of allergens/lipocalins in the eggs are not fully understood, they were speculated to be related to the defense of the eggs. That is, allergens/lipocalins may provoke serious anaphylaxis (such as tissue swelling, hypotension, itching and dermatitis) and blooding so that predators will not continuously eat the eggs, which is at least partially supported by the behavior characteristics of the animals that ate the eggs or were injected with the egg extract [13,14].

**CAP superfamily.** CAP superfamily includes the cysteine-rich secretory proteins (CRISPs), antigen 5, and pathogenesis-related 1 proteins superfamily and is also referred to as sperm-coating glycoprotein (SCP) protein family, because many proteins in this superfamily contain SCP domain [58]. The SCP domain-containing proteins are present in the venoms of cephalopods, cone snails, stinging insects, scorpions and spiders, and have the ability to inhibit cyclic nucleotide–gated channels, ryanodine receptor channels, and Ca^2+^ and K^+^ channels [57]. In this transcriptome analysis, five unigenes (Table S3) encode the proteins containing SCP domain. Such proteins were also discovered in the transcriptome of *L. tredecimguttatus* venom gland [8], indicating that the proteins with SCP domain are a class of common toxins endowing eggs and venom glands with toxicity, especially neurotoxicity.

**Plancitoxins I.** Plancitoxin I, a lethal factor from the crown-of-thorns starfish *Acanthaster planci*, is homologous with mammalian deoxyribonuclease II (DNase II), exhibits DNase activity responsible for the hepatotoxicity, and induces oxidative and endoplasmic reticulum stress associated cytotoxicity and apoptotic activities on A375.S2 cells [59,60,69,70,71]. In the eggs, two unigenes (Table S3) encode proteins showing major similarity with DNase II and the plancitoxin I from *Parasteatoda tepidariorum*. Therefore, the proteins in the eggs may be a plancitoxin I-like protein, exhibiting the lethal activity similar to the plancitoxin I in *A. planci*.

**Prokineticin/AVIT.** In the *L. tredecimguttatus* eggs, a unigene (Table S3) encodes a protein with prokineticin domain. According to the reports, prokineticin/AVIT is widely present in the venoms of reptiles, amphibians, spiders and ticks, and interacts with prokineticin receptors 1 and 2 to cause toxic effects, such as gastric smooth muscle contraction, hyperalgesia and even anorexogenic effect [57,61]. Thus, it can be speculated that during evolution of *L. tredecimguttatus*, this protein may be one of the potent toxins to protect eggs from the assaults by predators and contributes to the effective reproduction of *L. tredecimguttatus* in the nature.

**Chitinases and hyaluronidase.** Except the toxin-like proteins or enzymes described above, there are chitinases and hyaluronidase in *L. tredecimguttatus* eggs, which have been found in spider venoms [5,57]. According to the current knowledge, chitinases and hyaluronidase may act as “spreading factors” that facilitate the invasion of toxins by hydrolyzing chitin, extracellular matrix and other diffusive obstacles [46,65].

## 3. Discussion

In the present work, we made a comprehensive transcriptome study on *L. tredecimguttatus* eggs using Illumina RNA-Seq technology without prior genome information. To the best of our knowledge, this is the first transcriptome of spider eggs and is very helpful for understanding of the toxicity of the eggs. As is known, the venoms of spiders including *L. tredecimguttatus* are rich in a variety of peptides, proteins or enzymes with different biological activities, many of which are important components of the venom acting as “weapons” for the predation and defense of the spiders [5,7,28]. In this research, a batch of such bioactive molecules were identified in *L. tredecimguttatus* eggs at the transcriptome level, which not only demonstrated that molecular bases for the toxicity between the eggs and venom have a certain similarities, but also further supported the viewpoint that proteinaceous toxins convergently evolved. That is to say, numerous proteins and peptides have been convergently recruited into different tissues and organs of venomous animals to endow them with toxicity [57,72]. As shown in Table 3, the identified toxin-like proteinaceous components include hydrolases (metalloprotease, serine protease, phospholipase, phosphatase, cholinesterase, chitinase, hyaluronidase, etc.), protease inhibitors (Serpins), ion channel inhibitors (CAP superfamily proteins, serpins), allergens, plancitoxin-1 and so on. These components have the bioactivities to activate zymogen, degrade tissue proteins, inhibit ion channels, block neuromuscular transmission, provoke anaphylaxis, induce apoptosis or hyperalgesia, etc., and thus cooperatively attribute to the toxicity of the *L. tredecimguttatus* eggs. However, it is worthy of pointing out that although both venom and eggs of *L. tredecimguttatus* use some peptides, proteins and/or enzymes to participate in the construction of their toxicity basis, they share only a few identical proteinaceous components, and therefore have different toxicity mechanisms. This conclusion was supported by the fact that latrotoxins— typical *Latrodectus* proteinaceous venom toxins—were identified in the venom proteome [56] and venom gland transcriptome [8], but not in the egg proteome [15] and present egg transcriptome of *L. tredecimguttatus*.

Besides the above-mentioned toxin-like proteinaceous components, some peptides or proteins encoded by unigenes have typical structural characteristics of toxins (having signal peptide and many multiple cysteine residues) but were not counted into toxins, because they did not hit any known toxins in the existing databases, due to the lack of genome information and special database for *L. tredecimguttatus*. In our previous studies, latroeggtoxin-I to latroeggtoxin-IV were purified from the eggs and identified to be toxins [16,17,18]. However, they had not been identified to be toxins by BLAST analysis in the present transcriptome of the eggs, because the existing databases had not collected them as toxins when our current work was performed. In addition, even more unigenes had not been annotated due to lack of the special genome database of *L. tredecimguttatus*. It is speculated that at least part of those unigenes can encode toxic proteins and peptides that participate in the formation of molecular basis for the toxicity of the eggs. Therefore, the proteinaceous toxin profile in the *L. tredecimguttatus* eggs should be greater than we identified.

Bioinformatics analysis discovered that, of the14,185 unigenes annotated, only 280 unigenes (accounting for 1.97%) encode proteinaceous toxins. These results suggest that, although there were some other possible toxic components that had not been counted into toxins, the toxicity of the eggs is based on small part of the egg proteome, which may be partially explained by the fact that the proteins in the eggs are primarily involved in the nutrition storage, substance and energy metabolisms as well as their regulation during the development of the eggs into juvenile spiders [73], and their toxic effect is relatively less important. Besides, the synthesis of proteinaceous toxins is a process of high metabolic cost, which will take up extra biological resources used by other essential metabolic processes [74]. Therefore, in order to prevent the synthesis of proteinaceous toxins from disturbing essential cellular metabolism, the eggs have evolved strategies for synthesizing fewer numbers of proteinaceous toxins to exert maximum protective effects.

## 4. Conclusions

In the present study, we constructed the transcriptome of *L. tredecimguttatus* eggs without prior genome annotation using Illumina sequencing technology, which resulted in the identification of 14,185 unigenes, including 280 unigenes encoding proteins or peptides homologous to known proteinaceous toxins. Our present work not only provides an overview of the eggs’ proteinaceous toxin profile for comprehensively understanding of the molecular basis and action mechanism of egg toxicity, but also presents an overview of the protein composition and cellular processes in the eggs, which have laid a solid foundation for further studying and utilizing the proteinaceous components of the eggs in the future.

## 5. Materials and Methods

### 5.1. cDNA Library Construction, Sequencing and De Novo Assembly

Total RNA was isolated from *L. tredecimguttatus* eggs about 1–2 weeks before hatching of newborns using Trizol reagent (Invitrogen^TM^, Carlsbad, CA, USA) according to the manufacture’s protocol. Beads with oligo (dT) were used to purify poly (A) mRNA from the total RNA (Qiagen GmbH, Hilden, Germany). Following purification, the mRNA was randomly fragmented by heating to 94 °C in fragmentation buffer and the cleaved RNA fragments were used for the first strand cDNA synthesis using Superscript^TM^ III (Invitrogen^TM^, Carlsbad, CA, USA) and random hexamer primers. The second strand cDNA was synthesized by DNA polymerase I and RNase H. Double-stranded cDNAs were end repaired, A-tailed, and ligated to Illumina “PE adapters”. Discrete sized cDNA-adapter ligation products of 200–500 bp were selected by electrophoresis, purified from agarose gel slices using the QiaQuick Gel Extraction Kit (Qiagen, Hilden, Germany) and were enriched with PCR. Finally, the library was sequenced using Illumina/Solexa Hiseq^TM^ 2500. Given the influence of Solexa data error rate on the results, the raw reads were cleaned by removing adaptor sequences, empty reads and low quality sequences with software cutadapt 1.9 [75]. Clean reads were assembled de novo into unigenes by the software Trinity (version trinityrnaseq_r2013-02-25, Broad Institute of MIT and Harvard, Cambridge, MA, USA) with default parameters as previously described by Grabherr et al. [76]. Firstly, the clean reads were combined into contigs with a certain overlap length (κ-mer = 35). Using the read mate pairs, the resulting contigs were then joined into scaffolds. “N” was used to represent an unknown sequence between each pair of contigs to generate scaffolds. In order to obtain sequences that contain fewer Ns and can not be extended on either end, paired-end reads were used again for gap filling of scaffolds. Such sequences were defined as the transcripts in this work. To obtain distinct transcript clusters, the transcripts were classified using TGI Clustering tools [77]. For removing redundance from each cluster, the longest transcript in each cluster was chosen as the unigene.

### 5.2. Annotation and Identification of Proteinaceous Toxins

According to the function annotation of Nr database, the Blast2GO program was used to obtain GO annotations for the unigenes [78]. To map all of the annotated unigenes to GO terms in the database and calculate the number of unigenes associated with each term, the WEGO software was then used to perform the GO functional classification of all unigenes to view the distribution of gene functions of the species [79]. The KOG and KEGG pathway annotations were performed by Blastall software against Cluster of Orthologous Groups database and Kyoto Encyclopedia of Genes and Genomes database [80,81,82].

The hypergeometric distribution was used to analyze the enrichment of GO and KEGG pathways. The GO enrichment analysis of the 280 unigenes that were annotated to encode the proteins matching known toxins was conducted using GOseq software [83]. Hypergeometric test was used to find the significantly enriched GO terms comparing to the unigenes that were annotated in the available GO databases. The KEGG enrichment analysis of 280 unigenes was carried out by KOBAS software [84] and the unigenes that were annotated in KEGG databases were referred to as the universe of genes. The calculating formula used is as follows [85]:

$$P=1-\overset{m-1}{\underset{i=0}{\sum }}\frac{\left(\begin{array}{c} M\\ i \end{array}\right)\left(\begin{array}{c} N-M\\ n-i \end{array}\right)}{\left(\begin{array}{c} N\\ n \end{array}\right)}$$

For GO enrichment analysis, *N* is the number of all unigenes with GO annotation; *n* is the number of unigenes of interest in *N*; *M* is the number of all unigenes that are annotated to the certain GO terms; m is the number of unigenes of interest in *M*. For pathway enrichment analysis, *N* is the number of unigenes with KEGG annotation; *n* is the number of unigenes of interest in *N*; *M* is the number of unigenes annotated to specific pathways; m is the number of unigenes of interest in *M*. The corrected *p*-value ≤ 0.05 was chosen as the threshold value for significant enrichment.

To identify proteinaceous toxins, we performed the following annotation steps. Firstly, assembled unigenes were translated into protein sequences for all six reading frames. The six-frame translated protein sequences were used for local BLAST (version ncbi-blast-2.2.31^+^-win64, NCBI, Bethesda, MD, USA) against NR, Swiss-Prot, TREMBL and spider toxin database (ArachnoServer) with threshold values identity >30% and E-value < 10^−5^. Secondly, the conserved domains of proteins encoded by unigenes were also predicted using CCD and PFAM databases. The above results of BLAST search were parsed into a hit-sequence file. Finally, the hit-sequence file was searched with keywords “toxin”, “venom” and other toxin-like proteins reported in references [8,25,29,46,57] to mine putative proteinaceous toxins. If the results from different databases conflicted, a priority order of NR, Swiss-Prot, TREMBL and ArachnoServer was followed. In addition, special investigation of *L. tredecimguttatus* egg unigenes was carried out to find whether there were antimicrobial peptides in the eggs using the BLAST search against the antimicrobial peptide database (APD) with threshold values identity >30% and E-value < 10^−5^.

### 5.3. Other Bioinformatics Analyses

The signal peptide and ICK motif were predicted by Signal4.1 Server [86] and the konttin database [87]. The sequence alignment and phylogeny analysis were carried out using DNAman (version 8.0.8.789, Lynnon Biosoft, San Ramon, CA, USA) and MEGA3.1. (Biodesign Institut, Tempe, AZ, USA). To predict secondary structure of ICK peptides, the deduced amino acid sequence was submitted to the database: PORTER [88].

## Figures and Tables

**Table 1:** Summary of RNA-sequencing and read assembly.

Analysis of Read Assembly	Amount
Total number of reads	47,970,296
Total base pairs (bp)	5,836,590,233
Average read length (bp)	121
Total number of transcripts	69,684
Total number of unigenes	53,284
Average length of unigenes (bp)	738
Total number of unigenes >2000 bp in length	4376
Total number of unigenes annotated in at least one database	14,185

**Table 2:** Putative non-ICK toxins.

Sequence ID	Signal Peptide	Score (Bits)	E-Value	Identity	BLAST Annotation
comp24914_c0_seq1	N	64.7 (156)	8 × 10^−11^	34%	gb|AGA82764.1|toxin-like protein 14 precursor (*Urodacus yaschenkoi*)
comp6833_c0_seq1	N	92.0 (227)	4 × 10^−22^	56%	gb|ABR21046.1|venom toxin-like peptide-6 (*Mesobuthus eupeus*)
comp22890_c0_seq2	N	90.9 (224)	9× 10^−22^	56%	gb|ABR21046.1|venom toxin-like peptide-6 (*Mesobuthus eupeus*)
comp17051_c0_seq1	N	53.5 (127)	3× 10^−10^	32%	as: U_15_-SYTX-Sth1a||1459 Translation of a toxin from the spider *Scytodes thoracica* with unknown molecular target and function
comp25199_c0_seq1	Y	55.8 (133)	7× 10^−11^	32%	as: U_12_-SYTX-Sth1a||1449 Translation of a toxin from the spider *Scytodes thoracica* with unknown molecular target and function
comp21232_c0_seq1	Y	277 (709)	5× 10^−86^	98%	gb|ADV40303.1|cystatin-like protein (*Latrodectus Hesperus*)
comp20935_c0_seq1	Y	102 (255)	7× 10^−25^	40%	as: U_24_-ctenitoxin-Pn1a|sp:P84032|Toxin from venom of the spider *Phoneutria nigriventer* with unknown molecular target
comp213809_c0_seq1	Y	65.5 (158)	1× 10^−13^	29%	as: U_24_-ctenitoxin-Pn1a|sp:P84032|Toxin from venom of the spider *Phoneutria nigriventer* with unknown molecular target
comp27859_c0_seq1	Y	105 (263)	8× 10^−26^	44%	as: U_24_-ctenitoxin-Pn1a|sp:P84032|Toxin from venom of the spider *Phoneutria nigriventer* with unknown molecular target
comp96908_c0_seq1	Y	48.5 (114)	1× 10^−8^	35%	as: U_16_-aranetoxin-Av1a_1||2248 Toxin from venom of the spider *Araneus ventricosus* with unknown molecular target and function
comp5553_c0_seq1	Y	53.1 (126)	9× 10^−7^	32%	sp|Q8MTX1|TXCA_CAEEX U_3_-aranetoxin-Ce1a OS = *Caerostris extrusa* PE = 2 SV = 1

**Table 3:** Overview of the unigenes encoding toxin-like proteins or enzymes.

Name	Number of Unigenes	Percent of Unigenes (%)	Putative Activities
Metalloprotease	62	25	Activating proteinogen or zymogen [46,47]
			Degradating tissue to facilitate the spreading of toxins [46,47]
Serine protease	55	22.2	Activating proteinogen or zymogen [46,47,48]
			Degradating tissue to facilitate the spreading of toxins [46,47,48]
Phospholipase	41	16.5	Neurotoxicity, myotoxicity, etc. [46,47,48,49]
Serpin	30	12.1	Inhibiting degradation of proteinaceous toxins by protease [46,47,48]
			Acting on ion channels, e.g., K^+^ channel [50,51]
Phosphatase	18	7.3	Assisting the liberation of purines [52]
Cholinesterase	12	4.8	Blocking the neuromuscular transmission [53]
Allergen/Lipocalin	11	4.4	Provoking anaphylaxis [29,46,54,55,56]
			Disrupting hemostasis [57]
Chitinase	10	4	Degrading the chitin [57]
CAP superfamily	5	2	Modulating ion channels [57,58]
Plancitoxin-1	2	0.9	Inducing apoptosis [59,60]
Hyaluronidase	1	0.4	Enhancing tissue permeability to allow the spreading of toxins [46,47,48,57]
Prokineticin/AVIT	1	0.4	Inhibiting the feeding or inducing hyperalgesia [57,61]

## Supplementary Materials

The followings are available online at www.mdpi.com/2072-6651/8/12/378/s1, Figure S1: Statistical analysis of de novo assembly of *L. tredecimguttatus* egg unigenes. The length distributions of unigenes are shown, Figure S2: Multiple alignment analysis of predicted amino acid sequence of *L. tredecimguttatus* astacin-like metalloprotease. Amino acid identities are shaded in blue and conservative motif are designated by red boxes. Table S1: Information on GO Enrichment analysis for 280 unigenes encoding toxins, Table S2: Information on KEGG Enrichment analysis for 280 unigenes encoding toxins, Table S3: BLAST identification of the putative toxins of *L. tredecimguttatus* eggs.
