# Supplementary material for: Transcriptome Analysis to Understand the Toxicity of Latrodectus tredecimguttatus Eggs
Source: Toxins (Basel). 2016 Dec 20;8(12):378. doi: 10.3390/toxins8120378 (PMC5198572; doi:10.3390/toxins8120378)
Supplement: Supplementary file 1 [file toxins-08-00378-s001.zip › toxins-149093 supplementary proof/toxins-149093-supplementary-proof.docx]

Supplementary Materials: Transcriptome
Analysis to Understand the Toxicity of
*Latrodectus tredecimguttatus* Eggs

Dehong Xu and Xianchun Wang


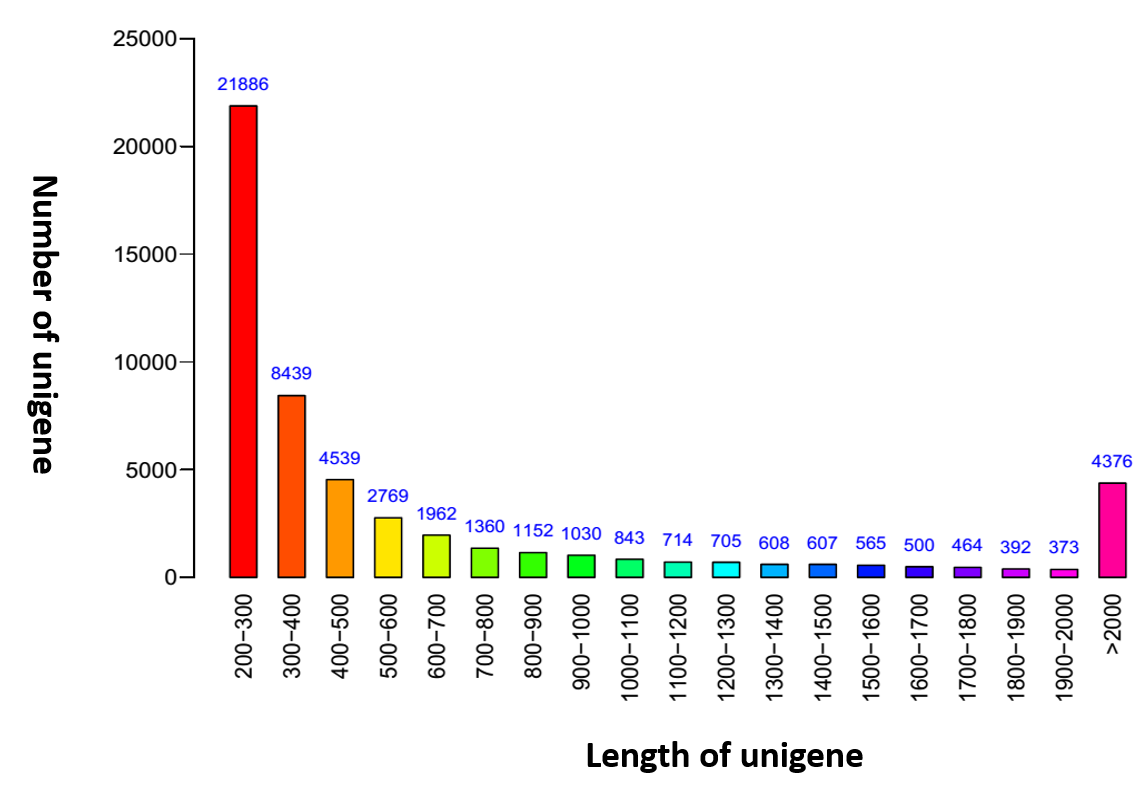


**Figure S1.** Statistical analysis of de novo assembly of *L. tredecimguttatus* egg unigenes. The length distributions of unigenes are shown.


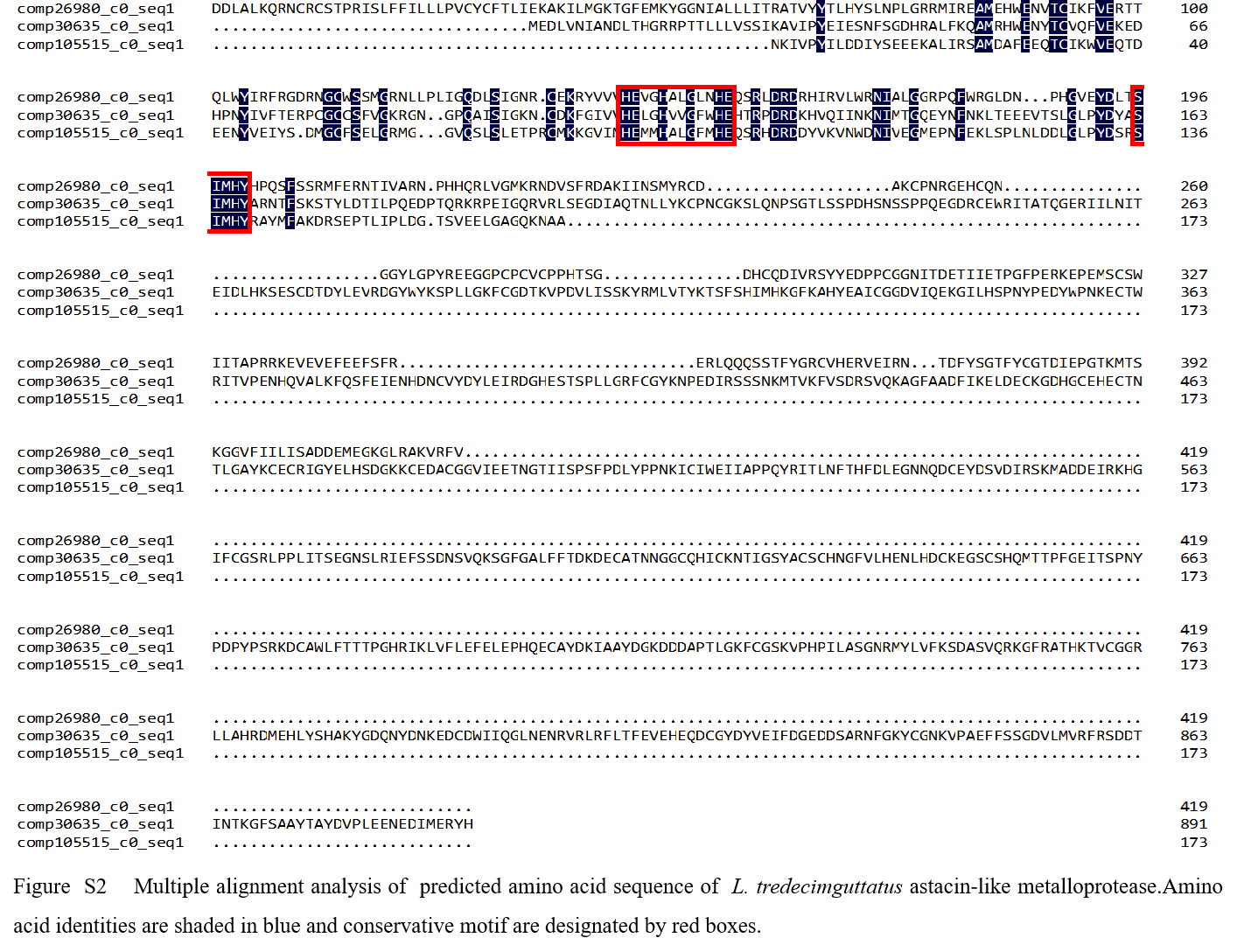


**Figure S2.** Multiple alignment analysis of predicted amino acid sequence of *L. tredecimguttatus* astacin-like metalloprotease. Amino acid identities are shaded in blue and conservative motif are designated by red boxes.
